# The role of the vasculature niche on insulin-producing cells generated by transdifferentiation of adult human liver cells

**DOI:** 10.1186/s13287-019-1157-5

**Published:** 2019-02-13

**Authors:** Irit Meivar-Levy, Fatima Zoabi, Gil Nardini, Eugenia Manevitz-Mendelson, Gil S. Leichner, Oranit Zadok, Michael Gurevich, Eytan Mor, Simona Dima, Irinel Popescu, Aviv Barzilai, Sarah Ferber, Shoshana Greenberger

**Affiliations:** 1 The Sheba Regenerative Medicine, Stem Cell and Tissue Engineering Center, Sheba Medical Center, Tel Hashomer, Israel; 2 Dia-Cure, Institute of Medical Scientific Research Acad. Nicolae Cajal, University Titu Maiorescu, Bucharest, Romania; 3 Sackler Faculty of Medicine, Tel-Aviv University, Tel-Aviv, Israel; 4 Department of Plastic Surgery, Sheba Medical Center, Tel Hashomer, Israel; 5 The Department of Dermatology, Sheba Medical Center, Tel Hashomer, Israel; 6 The Organ Transplantation Division, Schneider Children Medical Center, Petach Tikvah, Israel; 7 Center of Excellence in Translational Medicine – Fundeni Clinical Institute, Bucharest, Romania; 8 Center of Digestive Diseases and Liver Transplantation, Fundeni Clinical Institute, Bucharest, Romania

**Keywords:** Vasculature, Insulin-producing cells, Transdifferentiation, Bone marrow–derived mesenchymal stem cells, Endothelial colony-forming cells, Pancreatic transcription factors

## Abstract

**Background:**

Insulin-dependent diabetes is a multifactorial disorder that could be theoretically cured by functional pancreatic islets and insulin-producing cell (IPC) implantation. Regenerative medicine approaches include the potential for growing tissues and organs in the laboratory and transplanting them when the body cannot heal itself. However, several obstacles remain to be overcome in order to bring regenerative medicine approach for diabetes closer to its clinical implementation; the cells generated in vitro are typically of heterogenic and immature nature and the site of implantation should be readily vascularized for the implanted cells to survive in vivo. The present study addresses these two limitations by analyzing the effect of co-implanting IPCs with vasculature promoting cells in an accessible site such as subcutaneous. Secondly, it analyzes the effects of reconstituting the in vivo environment in vitro on the maturation and function of insulin-producing cells.

**Methods:**

IPCs that are generated by the transdifferentiation of human liver cells are exposed to the paracrine effects of endothelial colony-forming cells (ECFCs) and human bone marrow mesenchymal stem cells (MSCs), which are the “building blocks” of the blood vessels. The role of the vasculature on IPC function is analyzed upon subcutaneous implantation in vivo in immune-deficient rodents. The paracrine effects of vasculature on IPC maturation are analyzed in culture.

**Results:**

Co-implantation of MSCs and ECFCs with IPCs led to doubling the survival rates and a threefold increase in insulin production, in vivo. ECFC and MSC co-culture as well as conditioned media of co-cultures resulted in a significant increased expression of pancreatic-specific genes and an increase in glucose-regulated insulin secretion, compared with IPCs alone. Mechanistically, we demonstrate that ECFC and MSC co-culture increases the expression of CTGF and ACTIVINβα, which play a key role in pancreatic differentiation.

**Conclusions:**

Vasculature is an important player in generating regenerative medicine approaches for diabetes. Vasculature displays a paracrine effect on the maturation of insulin-producing cells and their survival upon implantation. The reconstitution of the in vivo niche is expected to promote the liver-to-pancreas transdifferentiation and bringing this cell therapy approach closer to its clinical implementation.

**Electronic supplementary material:**

The online version of this article (10.1186/s13287-019-1157-5) contains supplementary material, which is available to authorized users.

## Background

Islet transplantation is a promising procedure for the restoration of glycemic control in type 1 diabetes patients [1, 2]. However, this approach is limited by the shortage of donor-derived organs, the low rates of islet allograft survival, and the resulting high number of islets that are required per procedure. Among other cell replacement therapy approaches for diabetics is the option to reprogram adult extra-endocrine pancreatic tissues into functional glucose-regulated insulin-producing cells (IPCs). We demonstrated that adenoviral-mediated ectopic expression of pancreatic transcription factors (pTFs) induces transdifferentiation of adult liver cells into IPCs, both in vivo in mice and in vitro in human tissue [3–11]. The transdifferentiation protocol resulted in the generation of a heterogeneous population of cells in which the capacity for insulin production was restricted to a subpopulation of the human liver cells in the culture [10, 12]. Moreover, the survival of the implanted cells in vivo is low, either under the kidney capsule or subcutaneously [8, 13].

Pancreatic islets are extremely vascularized structures that have high levels of oxygen consumption. Although they form only 1–2% of the pancreatic mass, pancreatic islets receive 5–10% of the pancreatic blood with capillary density, which is ten times more than the surrounding exocrine pancreas [14]. Moreover, their proximity to the blood vessels plays an important role in the provision of non-nutritional signals to them, creating a vascular niche in which cross-talk between the endocrine β cells and endothelial cells is necessary to ensure proper pancreatic differentiation and β cell development and function [15–20]. While endocrine pancreatic cells express vascular endothelial growth factor A (*VEGF-A*), which is a crucial factor in maintaining the microvasculature of the islet [21–24], the vasculature produces several paracrine factors that modulate gene expression, proliferation, and the survival of β cells. Among these are thrombospondin-1 [25], transforming growth factor beta (*TGFβ*) [26], endothelin-1 [27], hepatocyte growth factor (HGF) [28], and connective tissue growth factor (CTGF) [29, 30].

Numerous reports indicate the key role that the endothelium has on pancreatic β cell differentiation and survival and the beneficial effect of the endothelial cells on the function of implanted islets [31–35]. In addition to the endothelial cells, intra-islet microvessels consist of one or more layers of perivascular cells [36, 37]. Studies have shown that the inclusion of human bone marrow mesenchymal stem cells (MSCs), which are able to differentiate into pericytes or smooth muscle-like cells when directly contacting endothelial cells, leads to increased vascularization [38] and survival of implanted pancreatic islets [39, 40].

Herein, we aim to analyze the effect of human bone marrow–derived MSCs and endothelial colony-forming cells (ECFCs) on the survival of IPCs, their function in vivo implantation, and the potential paracrine role of the MSCs and ECFCs on IPC maturation and function in vitro.

## Methods

### Human liver tissues and cultures

Liver tissues were used with the approval of the Sheba Medical Center Committee on Clinical Investigations (the institutional review board). All liver specimen donors or guardians on behalf of minors provided written informed consent for the collection of all samples and subsequent analyses. No donor organs were obtained from executed prisoners or other institutionalized persons. Tissue processing, isolation, and maintenance of cell cultures were described previously [10, 12].

### ECFC and MSC cultures

Human bone marrow–derived mesenchymal stem cells (MSCs) were isolated and propagated as previously described [38]. Cord-blood endothelial colony-forming cells (ECFCs) are a gift from Prof. Joyce Bischoff (Vascular Biology Program, Children’s Hospital Boston, and Harvard Medical School). Cells were isolated and phenotypically characterized as described [41, 42].

### Induction of TD in vitro

The recombinant adenoviruses, Ad-CMV-PDX1, Ad-CMV-NEUROD1, Ad-CMV-MAFA, and Ad-CMV-β-GAL, were cloned and propagated as described previously [10, 12].

Cells were plated in a low-glucose DMEM media (1 g/L glucose) supplemented with 2 mM l-glutamine, 10% serum, antibiotics, 10 mM nicotinamide, 20 ng/ml EGF, and 5 nM exendin-4. The cells were infected with 1000MOI Ad-CMV-PDX1 and 250MOI Ad-CMV-NEUROD1. After 48 h, the cells were harvested, counted, infected with 50MOI of Ad-CMV-MAFA, and re-plated as above. The infected cells were incubated for an additional 72 h before harvest.

### Mouse model

Animal procedures were approved by the Institutional Animal Care and Use Committee of Sheba Medical Center.

SCID-beige mice (8–9 weeks old, 18–19 g) were housed in an air-conditioned environment, under a 12-h light/dark cycle, and handled according to institutional animal welfare regulations. Generation of vascularized implants by co-implantation of endothelial cells and mesenchymal stem cells was previously described [38, 43, 44]. Shortly, IPCs (10^6^ cells) were mixed with ECFCs (10^6^ cells) and MSCs (10^6^ cells) in 200 μl of Matrigel (BD Biosciences, San Jose, CA) and injected subcutaneously into the mice. IPCs (10^6^ cells) without ECFCs and MSCs served as a control.

### Glucose tolerance test

Mice fasted for 6 h were injected intraperitoneally with glucose in saline at 2 mg/g body weight. Serum was collected for human C-peptide level analyses. The ultrasensitive Human C-Peptide ELISA kit (Mercodia, Uppsala) with 3% cross-reactivity to proinsulin but no cross-reactivity to mouse c-peptide was used according to the manufacturer’s instructions.

### Histology and staining

The implants were removed, fixed in 4% formaldehyde, embedded in paraffin, and stained as described in [8], using anti-human HLA-A (abcam ab52922, 1:100), anti-insulin (Sigma, clone K36 AC10, 1:75), and anti-human CD31 (Dako Cytomation, Clone JC70A, M0823, 1:50 dilution) antibodies. Analysis of HLA-positive cells was done by counting cells in random five × 40 fields from each implant. Histological analysis of microvessel density (MVD) was performed as described previously [38]; microvessel density was assessed by counting CD31 positive red blood cell–filled lumens. Values reported for each experimental condition correspond to the average MVD value ± standard error of the mean (SEM) obtained from all the individual implants.

Immunofluorescence: antibodies used in this study included guinea pig anti-insulin (1:400; Dako) and mouse anti-glucagon (1:800; Abcam). Fluorescent images were taken on a Nikon C1 confocal microscope at an original magnification × 40 as described [45].

### Cell culturing in the Transwell® system

IPCs were plated on the bottom of 12-well plates (10^5^ cells/well). After the cell adherence to the surface (3–4 h), the Transwell® inserts pre-coated with fibronectin were added to each well. The inserts were plated with ECFCs (10^5^ cells/insert) or MSCs (10^5^ cells/insert) or ECFCs /MSCs (1:1 ratio, total 10^5^ cells/insert) or with IPCs (10^5^ cells/insert) as control. After 72 h of co-culturing, the inserts were removed and the IPCs in the bottom of the wells were analyzed.

### Conditioned media

ECFCs and MSCs were plated alone or at 1:1 ratio at 7.5 × 10^4^/cm^2^ for 48 h in EBM2 media supplemented with 2 mM l-glutamine, 20% serum, and antibiotics. The conditioned media was collected, aliquoted, and frozen at − 20 °C. Heated conditioned media: the conditioned media was heated to 56 °C for 20 min in a water bath before supplemented to the cells.

### Isolation of ECFCs and MSCs from the co-cultures

ECFCs and MSCs were plated in six-well plates alone or at 1:1 ration at 2 × 10^4^/cm^2^. Human dermal fibroblasts, at the same density, were plated as a control. Five days later, cells were trypsinized and immune-separated using CD31 magnetic beads (Dynabeads®, Cat # 111.55D, Invitrogen) according to the manufacturer’s instructions.

### Insulin secretion in vitro

Glucose-regulated insulin secretion was measured by a radioimmunoassay kit (DPC, Los-Angeles, CA) as described previously [10, 12].

### Gene expression

RNA was prepared from cells using RNAeasy kit (Qiagen) and reverse-transcribed. Relative expression of indicated genes was measured by StepOne Real-Time PCR System using Fast SYBR Green Master Mix (Applied Biosystems). Relative quantitative analyses were performed according to the comparative CT method by using the arithmetic formula 2^−(ΔΔCt)^. The cDNA levels were normalized to β-ACTIN and GAPDH as the reference as described previously [10, 12]. Human pancreas total RNA (ThermoFisher) was used as a positive control.

### Statistical analyses

Statistical analyses were performed using two-sample Student’s *t* test assuming equal variances.

## Results

### Formation of the de novo blood vessels promotes the survival and function of IPCs in vivo

To analyze the effect of de novo vascularization on the maturation and function of IPCs, we co-implanted them with human bone marrow–derived MSCs and human cord-blood ECFCs in severe combined immunodeficiency (SCID)-beige mice. IPCs were generated by transdifferentiation of adult human liver cells that were induced by transcription factors, as was previously described [10]. MSCs and ECFCs were isolated and characterized [41, 42] (see also Additional file 1: Figure S1).

**Fig. 1:**
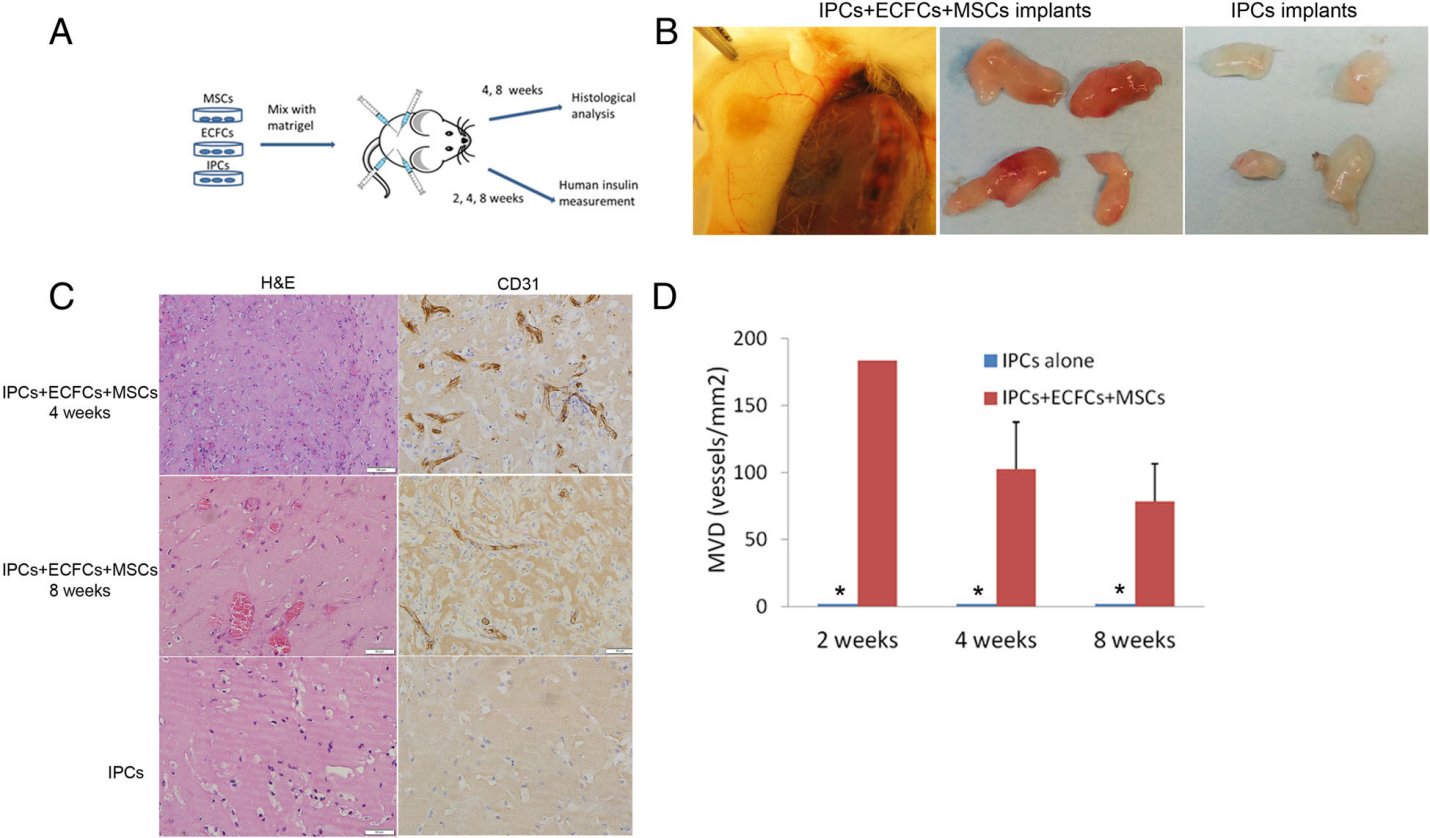
Co-implantation of MSCs, ECFCs, and IPCs promotes vascularization of the implants in vivo. SCID-beige mice were implanted subcutaneously with cells mixed with Matrigel, with four implants containing IPCs/ ECFCs/MSCs (1/1/1) implanted in each mouse (*n* = 8) or control implants of only IPCs (*n* = 6) as illustrated in **a**. **b** The implants were retrieved 4 to 8 weeks post implantation. **c** The implants were stained with H&E and anti-human CD31. Panels showing representative implants. Scale bar, 50 μm. **d** Microvessel density (MVD) was assessed by counting CD31-positive vessels. Values reported for each experimental condition correspond to the average MVD value ± standard error of the mean (SEM) obtained from *n* > 6 for 4 and 8 weeks

**Fig. 2:**
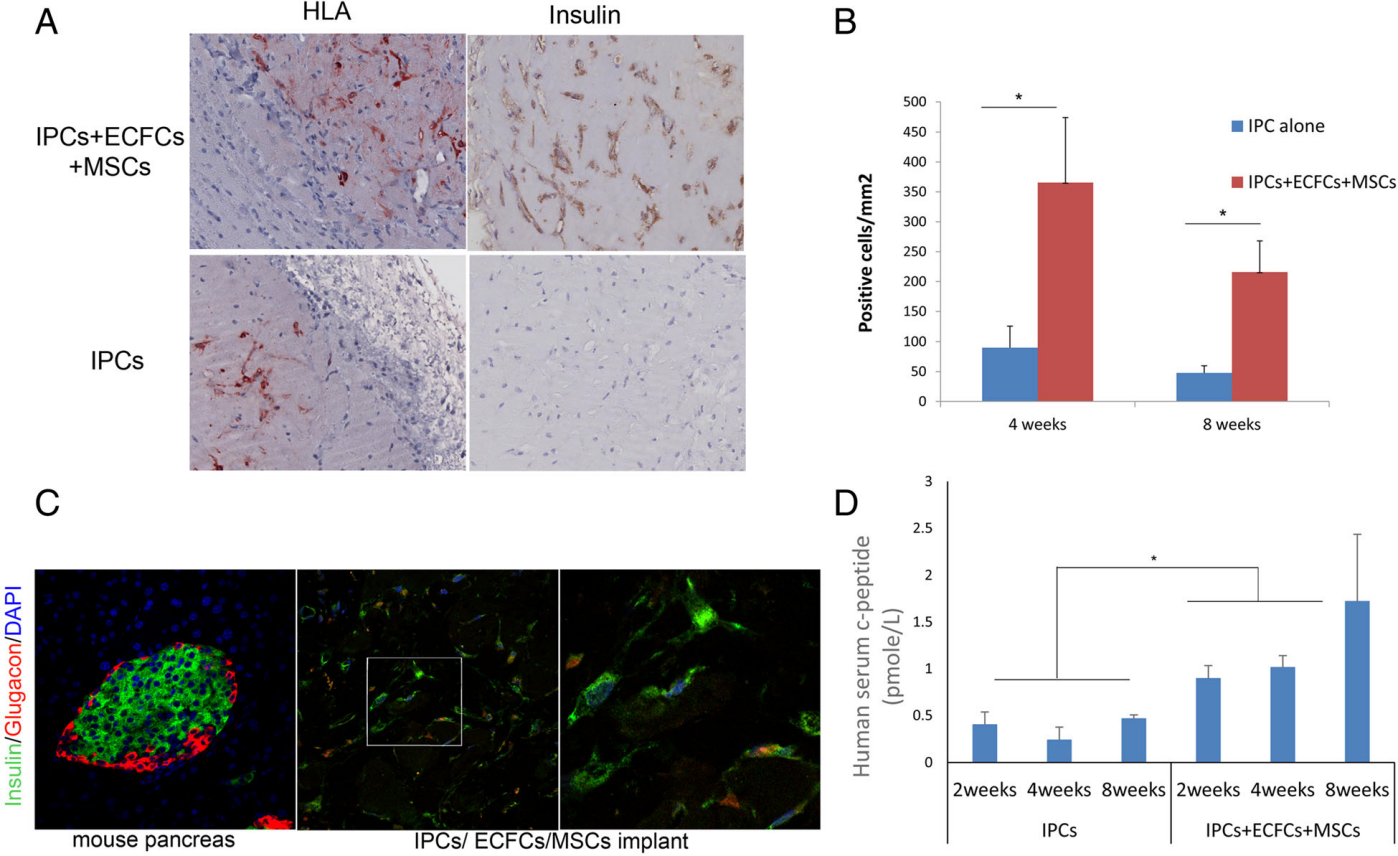
Human microvessels promote the survival and function of IPCs in vivo. **a** The implants retrieved after 4 weeks of implantation were stained using anti-HLA (**a**, upper panel) and anti-human insulin (**a**, lower panel) in the IPC/ECFC/MSC implants and the IPC implants. **b** Quantification of HLA-positive cells was done by counting cells in random five × 40 fields from each implant. Values reported for each experimental condition correspond to the average cell number ± standard error of the mean (SEM) obtained from *n* > 6 for 4 and 8 weeks, *P value < 0.05. **c** The IPC/ECFC/MSC implants were double stained for insulin (green) and glucagon (red). **d** Serum human c-peptide upon glucose stimulation was measured at 2, 4, and 8 weeks post implantation. The results are average and standard error (SE) for three to eight mice per group, at each time point, *P value < 0.05

Equal numbers of MSCs, ECFCs, and IPCs were mixed with Matrigel and implanted subcutaneously into SCID-beige mice: four implants per mouse (see study design in Fig. 1a and in [38]). As a control group, a similar number of IPCs were subcutaneously implanted in Matrigel but without MSCs and ECFCs. The implants were retrieved at 4 or 8 weeks post implantation. The retrieval rate of the implants containing the combination of MSCs, ECFCs, and IPCs was significantly higher than the rate for the implants containing IPCs alone (87.5% versus 41.6% after 8 weeks of implantation). Macroscopically, the cell combination implants appeared to be vascularized (Fig. 1b), while the Matrigel implants that contained IPCs were white or clear. In addition, microscopically, the combination implants showed significantly higher vascularization (Fig. 1c, d). Human CD31-positive vascular structures were seen only in the combination group (Fig. 1c, anti-human CD31, with no cross-reactivity to mouse CD31). At 8 weeks, reduction in the human CD31 staining was observed (Fig. 1c), suggesting that mouse vasculature protruded into the implants. The combination implants showed significantly higher cellularity; both vascular structures and dispersed single cells were positive for human leukocyte antigen (HLA) (Fig. 2a, b). Insulin-positive cells were significantly more abundant in the combination group, mainly in proximity to the blood vessels (Fig. 2a, c). In parallel to the increased number of insulin-positive cells that were detected in the mixed MSC, ECFC, and IPC cell implants (Fig. 2a, c), human blood insulin in the mice that were co-implanted with the cell mixture was higher than that in the mice that were implanted with only the IPCs, and this increased in accordance with the amount of time after implantation (Fig. 2d).

MSCs and ECFCs could affect the insulin production of the implanted IPCs by providing better oxygen and nutrient supplies and therefore promoting the survival of the cells. In addition, they could provide a preferred niche that supplies the implanted cells with growth factors needed for their maturation. To address the question of the beneficial effect of the blood vessels on the IPCs functionality, we established a controlled in vitro experimental system.

### ECFC/MSC co-culture promoted the pancreatic β cell-like maturation of IPCs in vitro

**Fig. 3:**
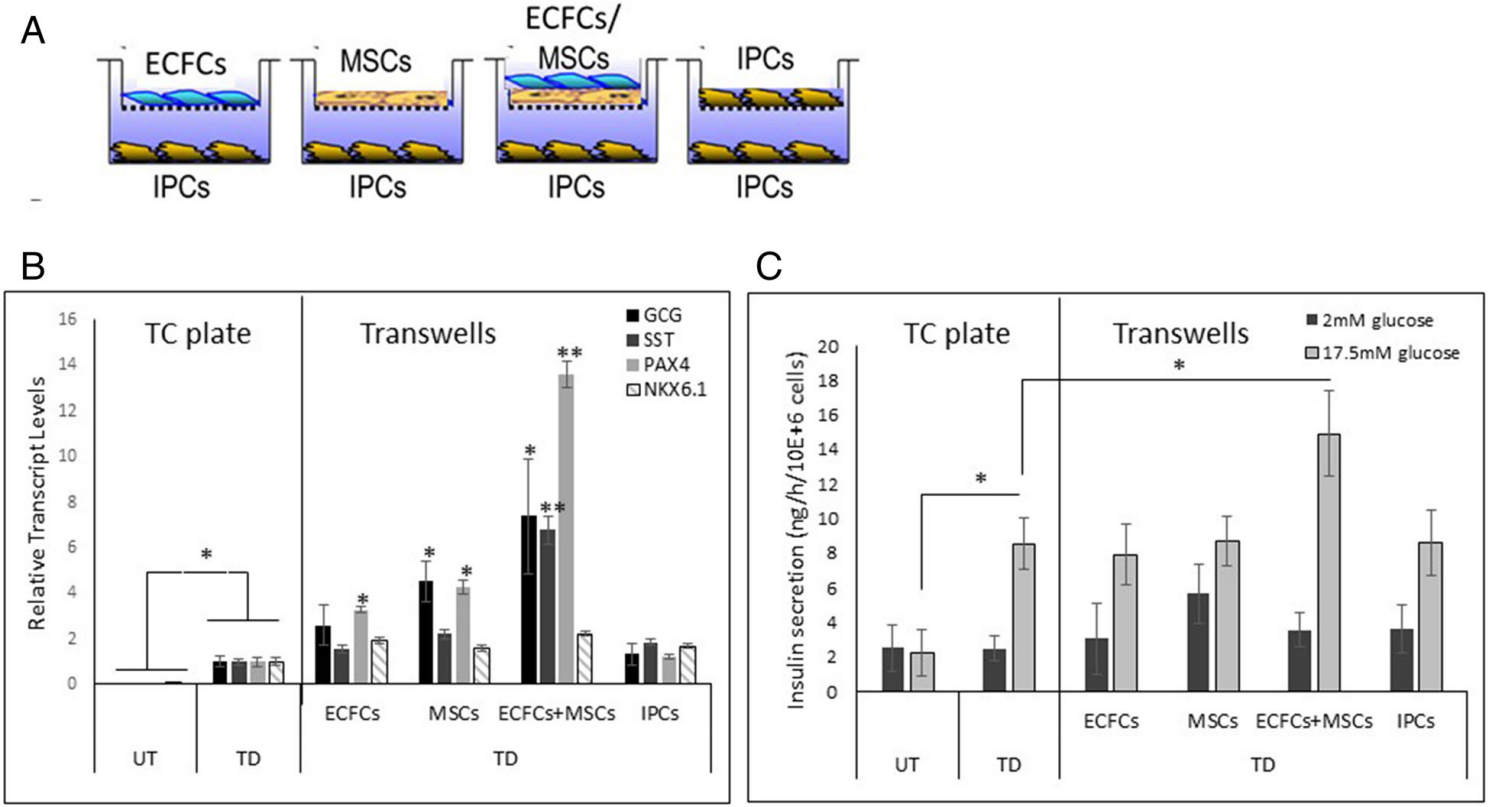
ECFC and MSC co-culturing in the Transwell® system promotes pTF induced liver-to-pancreas transdifferentiation in vitro. Induced IPCs were cultured on the bottom of the Transwell®; inserts were plated with ECFCs or MSCs or ECFCs + MSCs (1:1 ratio) or with IPCs as a control, as illustrated in **a**. After 3 days, the transdifferentiation efficiency of the cells was measured, as seen in **b**: the transcript levels of the pancreatic-specific genes are presented as average and SE for increases in the above cells treated with pTFs only, cultured in a regular 12 wells/plate (TC plate) and normalized to *β-ACTIN* levels, *P value < 0.05 and **P value < 0.01, respectively. **c** Glucose-regulated insulin secretion was measured using a specific human RIA kit (DPC). The secretion in the cells that were cultured in the Transwell® system was compared to cells cultured in a regular 12 wells/plate (TC plate). Results are presented as average and SE, *n* ≥ 12 from four independent repeats in different donors, *P value < 0.01 compared to cells treated with pTFs only, cultured in a regular TC plate treatment. The labeling below the graphs indicates the cell type cultured in the inserts of the Transwell® system

It has been shown that endothelial cells produce and secrete growth factors, cytokines, and other molecules with paracrine effects that promote pancreatic development and function [15–20]. To analyze the individual and concerted paracrine effect of ECFCs and MSCs on liver-to-pancreas transdifferentiation, we cultured the transdifferentiated IPCs in the lower compartment of a Transwell® system, with ECFCs, MSCs, and their combined co-culture in the upper compartment (Fig. 3a). The IPCs in the lower compartment were analyzed for alterations in the β cell-like phenotype (Fig. 3b) and function (Fig. 3c) because of their differential exposure to the distinct populations of cells in the upper compartment. The nutrient supply (media and serum) in all treatments was identical.

Either ECFCs or MSCs slightly increased the pancreatic gene expression of the IPCs (Fig. 3b). However, only ECFC and MSC co-culture in the upper compartment resulted in a significant increase in glucose-regulated insulin secretion and a robust increase in the activation of pancreatic-specific genes, such as *GCG*, *SST*, and *PAX4* expression (Fig. 3b, c). Thus, our data suggest that the combination of ECFCs and MSCs displays a specific synergistic paracrine effect on the maturation of IPCs and β cell-like function.

### Co-culturing ECFCs and MSCs promotes the expression of growth factors

**Fig. 4:**
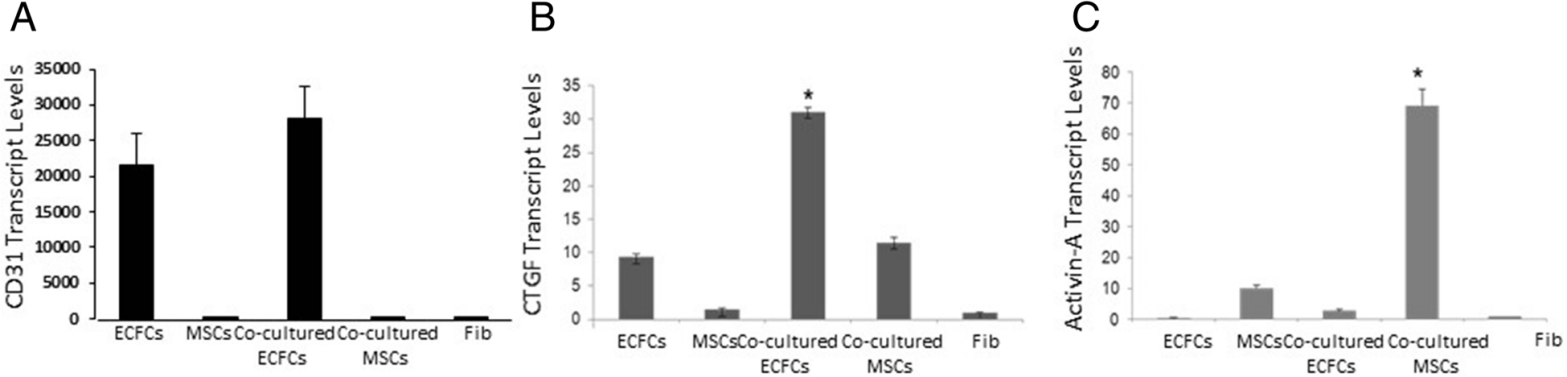
Co-culturing ECFCs and MSCs promotes the expression of specific growth factors. MSCs and ECFCs were cultured separately or co-cultured at a 1:1 ratio. Five days later, cells from the co-culture were immune-separated. *CD31* (**a**), *CTGF* (**b**), and *ACTIVIN-A* (**c**) transcript levels were analyzed. The results were normalized to *GAPDH* gene expression. The results are presented as average and SE, *n* = 2 independent repeats in triplicates, with *P value < 0.01 compared to human skin fibroblasts serving as a negative control

The data presented in Fig. 2 suggest that there may be a bilateral effect between the ECFCs and MSCs, which could modulate the pancreatic β cell-like function of the IPCs. The ECFCs and MSCs may have a unique bilateral effect on each other, where only their co-culture rather than any individual culture affects the β cell-like maturation of the IPCs. To gain an initial insight into the growth factors, cytokines, and other molecules that may mediate the paracrine effects of the conditioned media, we analyzed the effects of the ECFC and MSC co-culturing on the expression of specific growth factors known to be significant for pancreatic cell differentiation. ECFC and MSC cells that had been co-cultured for 5 days were mixed with beads coated with anti-CD31 that specifically bound the ECFCs and separated them from the MSCs. The two isolated cell types were analyzed separately for alterations in gene expression. The efficiency of cell separation was validated by the specific *CD31* gene expression that was restricted to the bound ECFCs and was absent from the eluted MSCs (Fig. 4a). ECFC and MSC co-culturing resulted in an increased *CTGF* gene expression in the ECFCs and *ACTIVIN*-A (*INHBA*) in the MSCs (Figs. 4b and 4c).

This initial insight suggests that co-culturing ECFCs with MSCs results in significant changes in the expression of the growth factors, cytokines, and other molecules that upon protein secretion could account for the paracrine effects of the conditioned media that are at low levels or absent in each individual type of cell when cultured alone.

### The conditioned media of the ECFCs and MSCs promotes the pancreatic differentiation

**Fig. 5:**
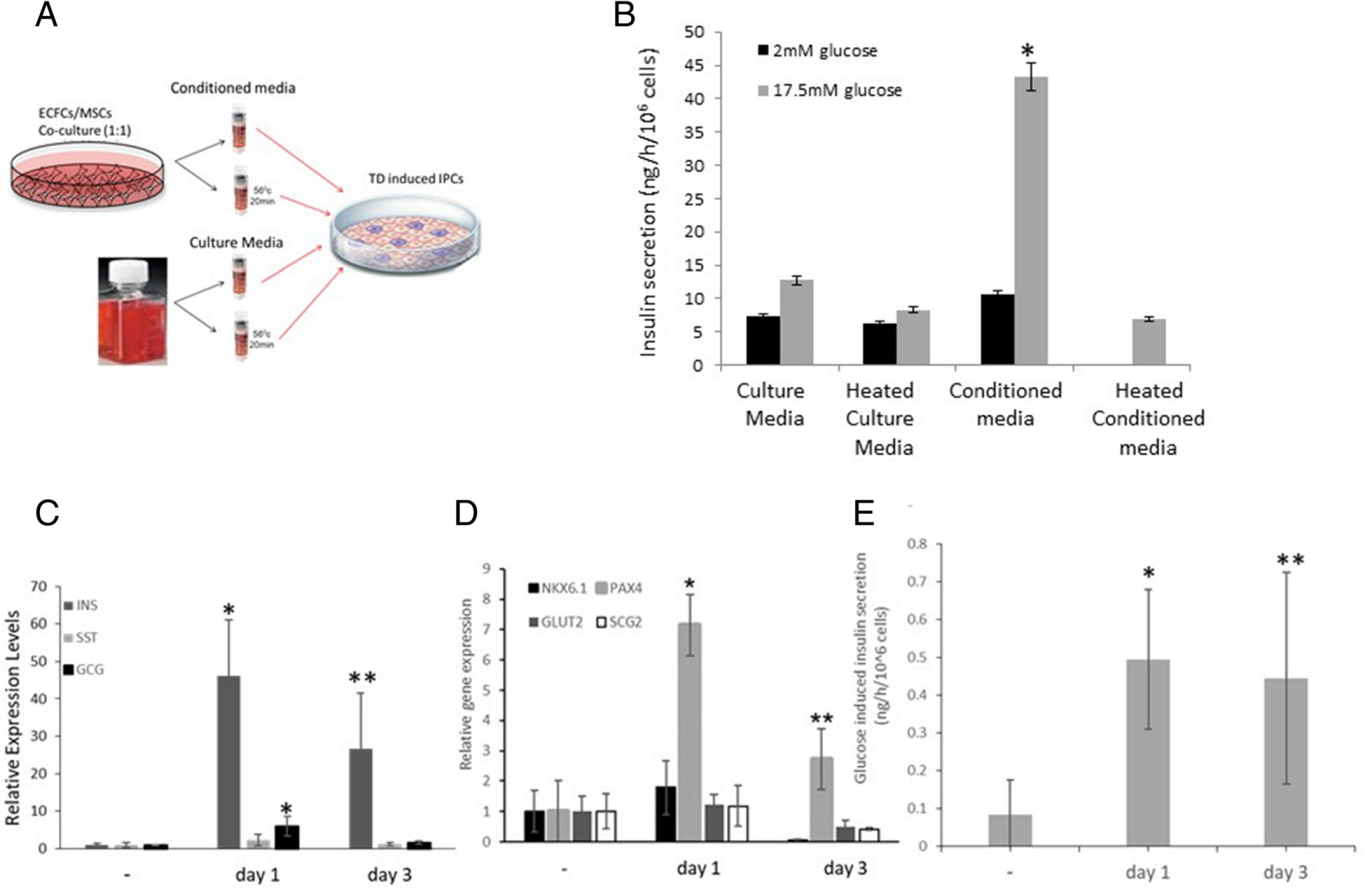
Conditioned media promotes pancreatic differentiation during the TD process. Conditioned media was collected after 48 h of co-culturing the ECFCs and MSCs (1:1 ratio). The media was added to the liver cell that was undergoing transdifferentiation either when fresh or after being heated to 56 °C (1:1 ratio with TD media), as illustrated in **a**. **b** The IPCs were analyzed for glucose-regulated insulin secretion. Insulin levels were analyzed using a specific human RIA kit (DPC) and compared to cells cultured in TD media (fresh or heated). Results are presented as average and SE for increases above secretion at low glucose in TD media, *n* ≥ 8 from three independent repeats in different donors, *P value < 0.01 compared to control treatment (**c**–**e**). The conditioned media was added (1:1 ratio with TD media) at day 1 or day 3 of the TD process, and the pancreatic phenotype was analyzed by specific pancreatic gene expression (**c**, **d**) and glucose-induced insulin secretion (**e**). Transcript levels of the indicated genes are presented as average and SE for increases above cells treated with pTFs alone and normalized to β-actin levels. *N* = 3–4 independent repeats in different donors, *P value < 0.01 and **P value < 0.05 compared to control treatment. Insulin levels were analyzed using a specific human ELISA kit (ALPCO) and compared to cells cultured in TD media. The results are presented as average and SE normalized to cell number, *n* ≥ 8 from three different experiments, *P < 0.01 and **P < 0.05 compared to control treatment

To verify whether the promoting effects that the ECFCs and MSCs have on the transdifferentiation process do in fact occur through compounds secreted from the cells, we co-cultured the ECFCs and MSCs for 48 h (1:1 ratio, as in Fig. 3) and collected the conditioned media, as illustrated in Fig. 5a. Supplementing the conditioned media of the ECFCs and MSCs to the transdifferentiation protocol promoted the process as indicated by the significant increase in glucose-regulated insulin secretion (Fig. 5b). Moreover, the conditioned media could be frozen and thawed without losing its activity (data not presented). However, conditioned media that was heated to 56 °C (20 min) lost its beneficial effect on the reprogramming process.

The reprogramming of liver cells toward IPCs is a sequential and temporally controlled process that resembles pancreatic differentiation [10]. While *PDX1* and *NEUROD1* activate the transdifferentiation process required for the initiation of the reprogramming process (days 1–3), *MAFA* promotes IPC maturation and is therefore required at later stages (days 3–6). We sought to analyze whether the “vasculature-derived conditioned media” is mainly efficient at the initial steps of pancreatic differentiation (days 1–3) or during IPC maturation (days 3–6). We either added the conditioned media from the co-cultured ECFCs and MSCs at day 1 together with *PDX1* and *NEUROD1* or on day 3 together with *MAFA*, or we added the conditioned media at both time points.

The greatest effect of the conditioned media on the activation of the pancreatic phenotype occurred when it was supplemented on the first day with the ectopic expression of *PDX1* and *NEUROD1* (Fig. 5c–e); insulin gene expression and *PAX4* gene expression were significantly higher when the conditioned media was added on the first day. Glucose-induced insulin secretion was similarly elevated regardless of the stage of conditioned media supplementation (Fig. 5e).

These data suggest that the transdifferentiation process may benefit from the paracrine effects of ECFCs and MSCs during the initial stages of the process, although some promoting effects may also occur during the entire process (Fig. 5c–e).

## Discussion

The present study demonstrates the beneficial role the vasculature plays in promoting pancreatic β cell-like differentiation and the functioning of reprogrammed human liver cells. The mixture of ECFCs and MSCs improved the survival and insulin secretion of the transdifferentiated IPCs in vivo. Moreover, using an isolated in vitro model, we showed that ECFCs and MSCs display a bilateral effect toward each other, since only their co-culture displayed a synergistic, paracrine effect on the expression of pancreatic-specific genes and on the glucose-regulated insulin secretion of the IPCs. This paracrine effect was potentially mediated by the secreted heat-sensitive proteins and other compounds. Mechanistically, we demonstrated that the proximity of the ECFCs and MSCs to each other in the co-culture was obligatory for the increase of the expression of factors such as *CTGF* and *ACTIVIN*-A, known to play key roles in pancreatic differentiation.

Our approach included the co-implantation of MSCs and ECFCs with IPCs. A combination of several types of endothelial cells and mural cells has been previously used for islet transplantation, such as HUVEC and MSCs [46] and HUVEC and fibroblasts [40]. Several advantages are associated with using ECFC and bone marrow–derived MSCs. First, both cell types can be isolated from the bone marrow of an adult donor. This enables the autologous transplantation of all three types of cells without immune rejection. Second, both cell types are highly proliferative cells in vitro*,* and their combination has been shown to form long-lasting, perfused microvascular networks that alleviate ischemia in models of hind limb ischemia and myocardial infarction [47]. Lastly, these two cell types were shown to have reciprocal interactions in our study (Fig. 4). Previously, it was demonstrated that ECFCs modulated the regenerative potential of the MSCs by paracrine secretion of angiocrine factors, whereas the MSCs modulated the ECFCs’ immunogenicity [48].

The identity of the endothelial cells and mural cells may also have affected the combination of the secreted cytokines and growth factors found in the conditioned media and, as a result, affected the maturation of the IPCs. Our in vitro assays clearly indicated that co-culturing ECFCs with MSCs resulted in increasing the expression of the analyzed *CTGF* and *ACTIVIN*-A genes because the cells influenced each other. The specific paracrine role of *CTGF* and *ACTIVIN*-A in promoting the transdifferentiation process should be further investigated by direct supplementation of *CTGF* and *ACTIVIN*-A proteins and by blocking these factors’ activity using neutralizing antibodies. The combination of the secreted cytokines and growth factors from co-culturing ECFCs with MSCs promoted the transdifferentiation process, mainly at the initial step of reprogramming.

Different combinations of secreted cytokines and growth factors are expected to affect the reprogramming process in different ways: some may promote the maturation of IPCs, while others may inhibit the process. The secreted cytokines and growth factors in the conditioned media could promote the reprogramming process by affecting the epigenome or by activating specific pancreatic gene expression or induction of post-transcriptional modifications. Our data suggest that the promoting effect of the conditioned media was potentially attributable to the post-transcriptional stages of insulin production, since it had no effect on ectopic insulin promoter activity (see Additional file 2: Figure S2).

## Conclusions

Taken together, our data suggest the importance of the vasculature niche on the therapeutic outcome of reprogrammed IPCs in the treatment of diabetes. The vasculature niche not only promotes implant survival in vivo, it also provides the signals required by the reprogrammed cells to differentiate toward functional, pancreatic insulin-producing cells. Indeed, the vasculature niche is expected to play a crucial role in many regenerative medicine approaches for degenerative diseases in general.

## Additional files

Additional file 1: **Figure S1.** Human ECFCs and MSCs characterization. ECFCs derived from cord-blood present a typical cobble-stone morphology (A). Cells expressed the endothelial markers, vWF (IMF; B) and CD31, and were negative for αSMA (FACs; C). BM derived MSCs present a typical fibroblast like morphology, the cells have multi-potential differentiation activity, evident by osteogenic (E) and adipogenic (F) differentiation. D-negative control (growth medium). H. bmMSCs were analyzed by single staining of negative panel markers (CD3, CD14, CD34 and CD45) and positive panel markers (CD73, CD90, CD105 and CD166). (JPG 132 kb)

Additional file 2: **Figure S2.** Conditioned media from ECFCs and MSCs do not increase activation of ectopic insulin promoter in IPCs pancreas in vitro. The conditioned media was added (1:1 ratio with TD media) at day 1 or day 3 of the TD process or at both days. Activation of ectopic insulin promoter was analyzed by infecting the cells at day 3 of the TD with Ad-RIP-Luciferase. The levels of activation were measured at day 6 by the luciferase activity and was compare to the expression levels of control untreated cells and TD alone. Results are presented as average and SE *n* = 3 independent repeats in different donors *P < 0.005 compared to control levels. (JPG 32 kb)

## Abbreviations

CTGF: Connective tissue growth factor
ECFCs: Endothelial colony-forming cells
IPCs: Insulin-producing cells
MSCs: Mesenchymal stem cells
MVD: Microvessel density
pTFs: Pancreatic transcription factors
