# Supplementary figures and images for: The role of the vasculature niche on insulin-producing cells generated by transdifferentiation of adult human liver cells
Source: Stem Cell Res Ther. 2019 Feb 13;10:53. doi: 10.1186/s13287-019-1157-5 (PMC6373031; doi:10.1186/s13287-019-1157-5)

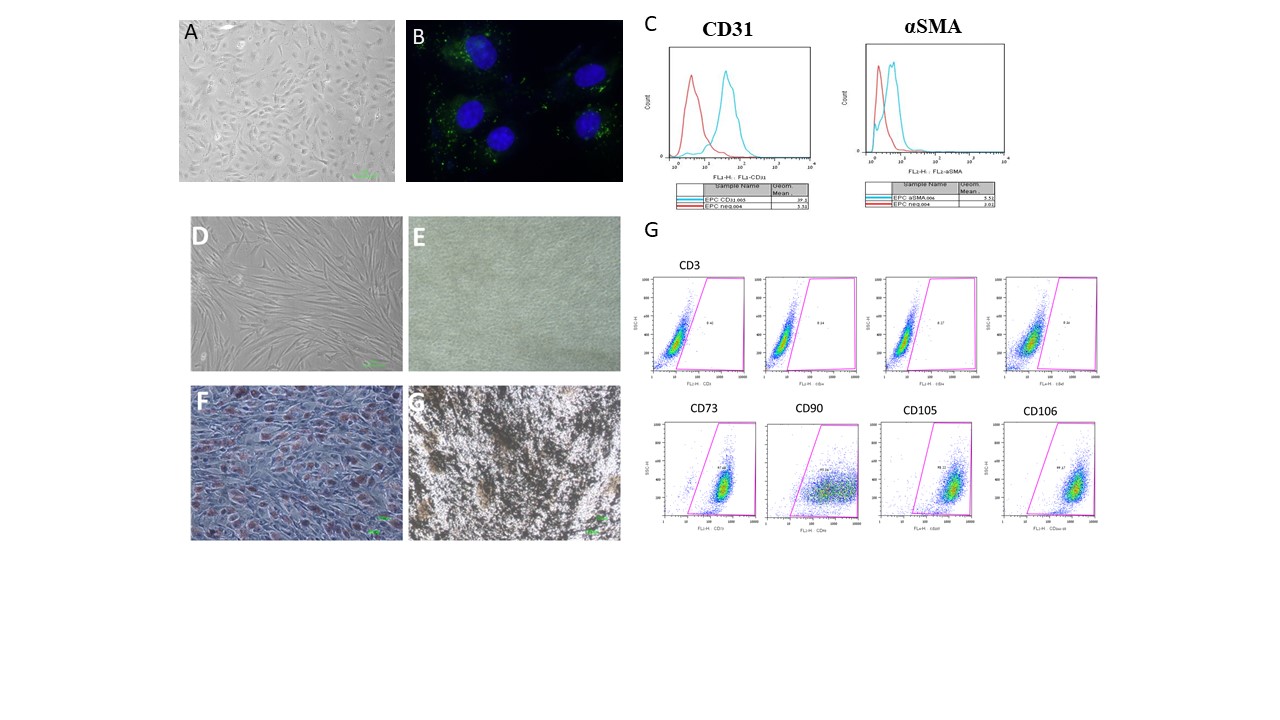

Supplement: Supplementary file 1 — Figure S1. Human ECFCs and MSCs characterization. ECFCs derived from cord-blood present a typical cobble-stone morphology (A). Cells expressed the endothelial markers, vWF (IMF; B) and CD31, and were negative for αSMA (FACs; C). BM derived MSCs present a typical fibroblast like morphology, the cells have multi-potential differentiation activity, evident by osteogenic (E) and adipogenic (F) differentiation. D-negative control (growth medium). H. bmMSCs were analyzed by single staining of negative panel markers (CD3, CD14, CD34 and CD45) and positive panel markers (CD73, CD90, CD105 and CD166). (JPG 132 kb) [file 13287_2019_1157_MOESM1_ESM.jpg]

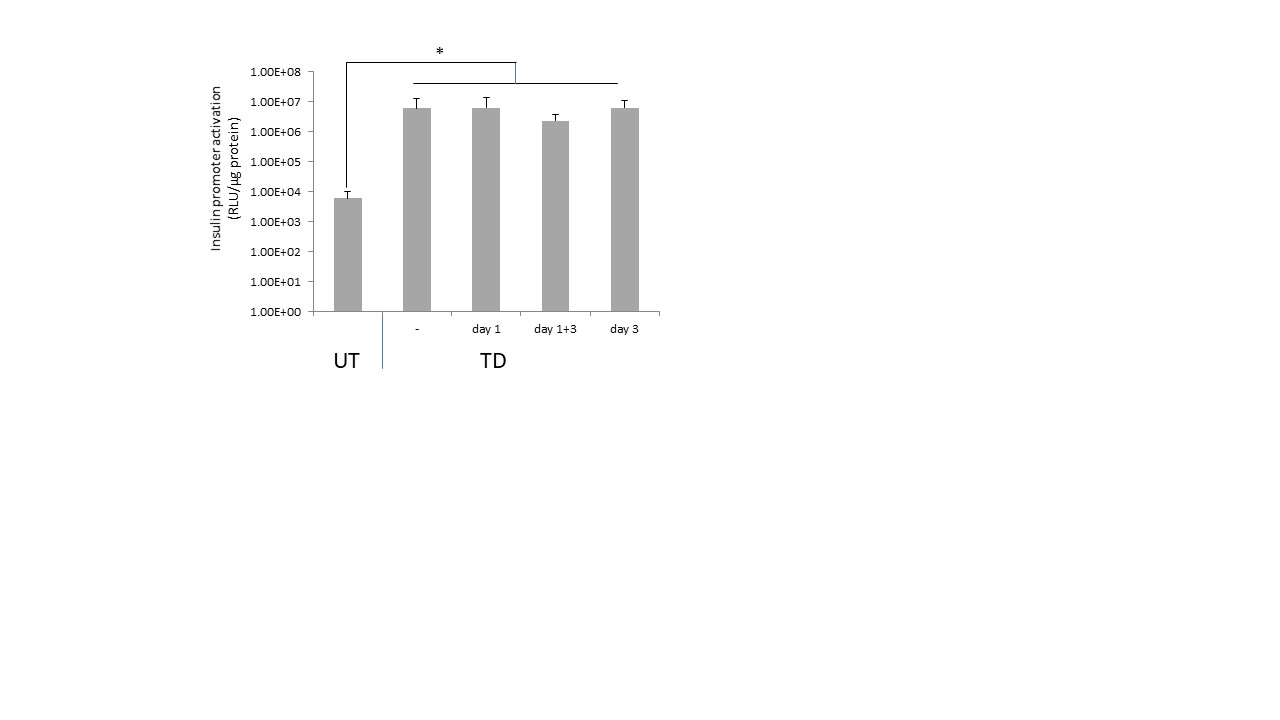

Supplement: Supplementary file 2 — Figure S2. Conditioned media from ECFCs and MSCs do not increase activation of ectopic insulin promoter in IPCs pancreas in vitro. The conditioned media was added (1:1 ratio with TD media) at day 1 or day 3 of the TD process or at both days. Activation of ectopic insulin promoter was analyzed by infecting the cells at day 3 of the TD with Ad-RIP-Luciferase. The levels of activation were measured at day 6 by the luciferase activity and was compare to the expression levels of control untreated cells and TD alone. Results are presented as average and SE n = 3 independent repeats in different donors *P < 0.005 compared to control levels. (JPG 32 kb) [file 13287_2019_1157_MOESM2_ESM.jpg]
